# Effectiveness of monovalent rotavirus vaccine in the Philippines

**DOI:** 10.1038/s41598-018-32595-9

**Published:** 2018-09-24

**Authors:** Anna Lena Lopez, Jedas Veronica Daag, Joel Esparagoza, Joseph Bonifacio, Kimberley Fox, Batmunkh Nyambat, Umesh D. Parashar, Maria Joyce Ducusin, Jacqueline E. Tate

**Affiliations:** 1 Institute of Child Health and Human Development, University of the Philippines Manila, Manila, 1000 Philippines; 2 Democrito O. Plaza Memorial Hospital, Prosperidad, 8500 Philippines; 3 Department of Virology, Research Institute for Tropical Medicine, 1781 Muntinlupa, Philippines; 4 Expanded Programme on Immunisation, Regional Office for the Western Pacific, World Health Organization, Manila, 1000 Philippines; 5 Division of Viral Diseases, Centers for Disease Control and Prevention, Atlanta, GA 30333 USA; 6 Disease Prevention and Control Bureau, Department of Health, Manila, 1003 Philippines; 7 Present Address: Global Immunization Division, Centers for Disease Control and Prevention, Atlanta, GA 30329 USA

## Abstract

Rotavirus (RV) is an important cause of diarrheal disease particularly in children aged under 5 years. Monovalent RV vaccine (RVV) was selectively introduced in 2012 in the Philippines and in July 2014 was introduced in the public health program of a province. Two RVV doses are recommended at 6 and 10 weeks of age. We conducted a test negative case-control evaluation to assess the effectiveness of RVV when given in a routine public health program in the Philippines. From September 2014 to August 2017, 967 children aged <5 years were hospitalized with diarrhea and of these, we enrolled 600 who were eligible to have received RVV and provided stool specimens for testing. Among children ≥8 months of age who were age-eligible to have received RVV, at least one dose of RVV had an adjusted vaccine effectiveness (VE) against RV hospitalization of 60% (95% confidence interval, CI: 24%, 79%), and against severe rotavirus diarrhea, VE was 64% (95% CI: 11%, 85%). These findings support the introduction of RVV into routine public health use in the Philippines. However, other factors such as costs, cost-effectiveness and operational issues must be considered prior to adoption of the vaccine into the countries’ public immunization program.

## Introduction

Diarrheal disease is a significant cause of mortality and morbidity in young children in the developing world. Diarrheal diseases also adversely affect long term growth and development^1^ providing further impetus for the use of appropriate preventive measures, including vaccination against diarrheal diseases.

Rotavirus (RV) is the most common cause of diarrhea globally and several vaccines are now internationally licensed and prequalified by the World Health Organization (WHO)^2,3^. Protection afforded by rotavirus vaccine (RVV) against severe RV diarrhea has been shown to vary depending on the country’s level of development^4–6^. In Asia, few countries have introduced RVV. Earlier results from high income countries of Asia in Hong Kong^7^ and Japan^8^, where RVV are self-financed, revealed RVV effectiveness of 89% and 70%, respectively against diarrheal hospitalization. However, a cluster-randomized study conducted in Bangladesh, a low income country, revealed that the monovalent RVV effectiveness was 41.4%^9^.

RVV was first introduced in the Philippines’ national immunization program in 2012, targeting children who belonged to the poorest quintile. However, there were problems in identifying the children, there were questions on the economic sustainability of the program and due to the varying levels of protection provided by the vaccine, policymakers in the Philippines requested that an effectiveness evaluation be conducted to assess the RV vaccine’s relevance for inclusion in the country’s Expanded Programme on Immunization (EPI). The vaccination strategy was changed limiting RVV use in the area where an effectiveness evaluation was conducted.

## Results

**Figure 1:**
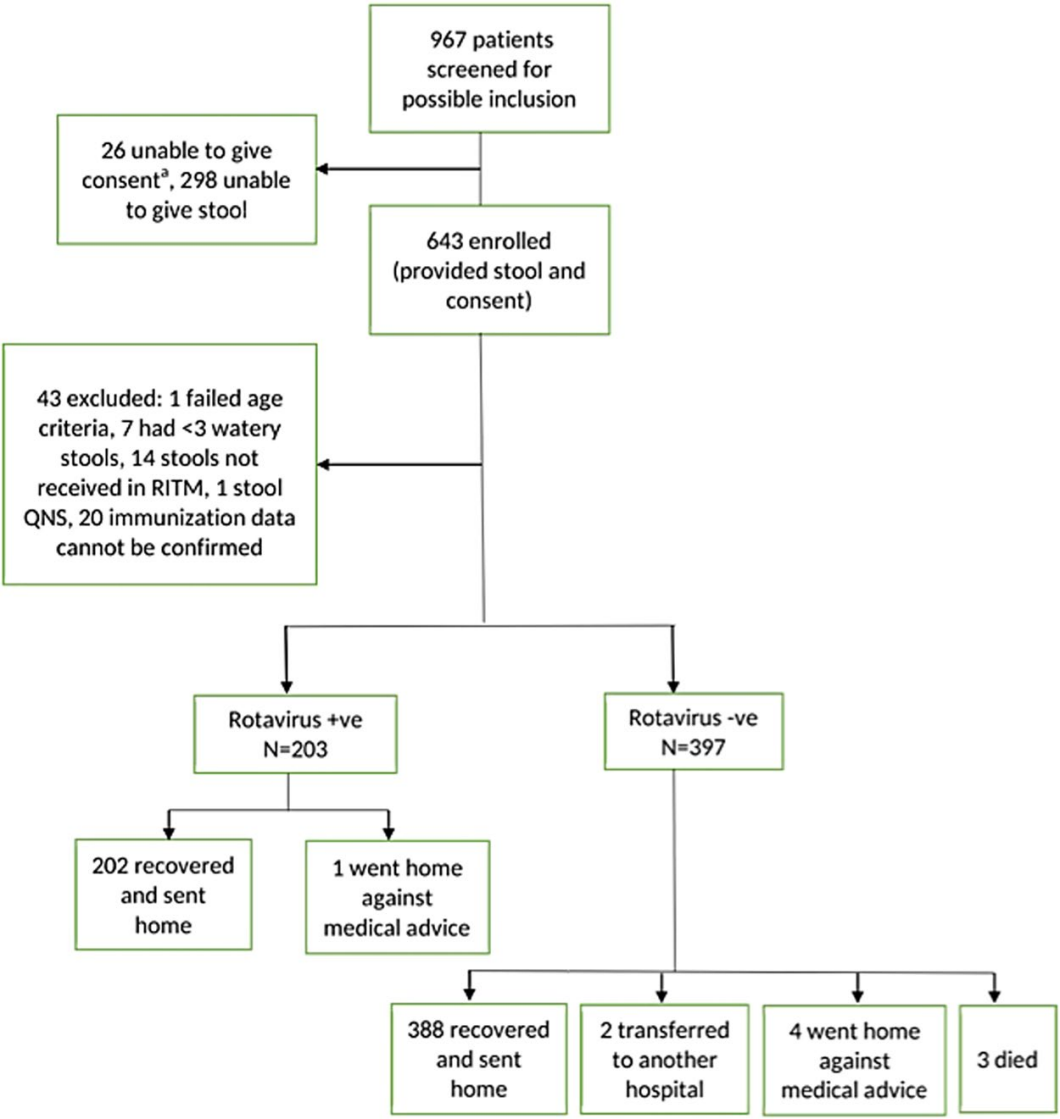
Flow of subjects in the study. ^a^Unable to get consent because these children arrived on weekends.

**Figure 2:**
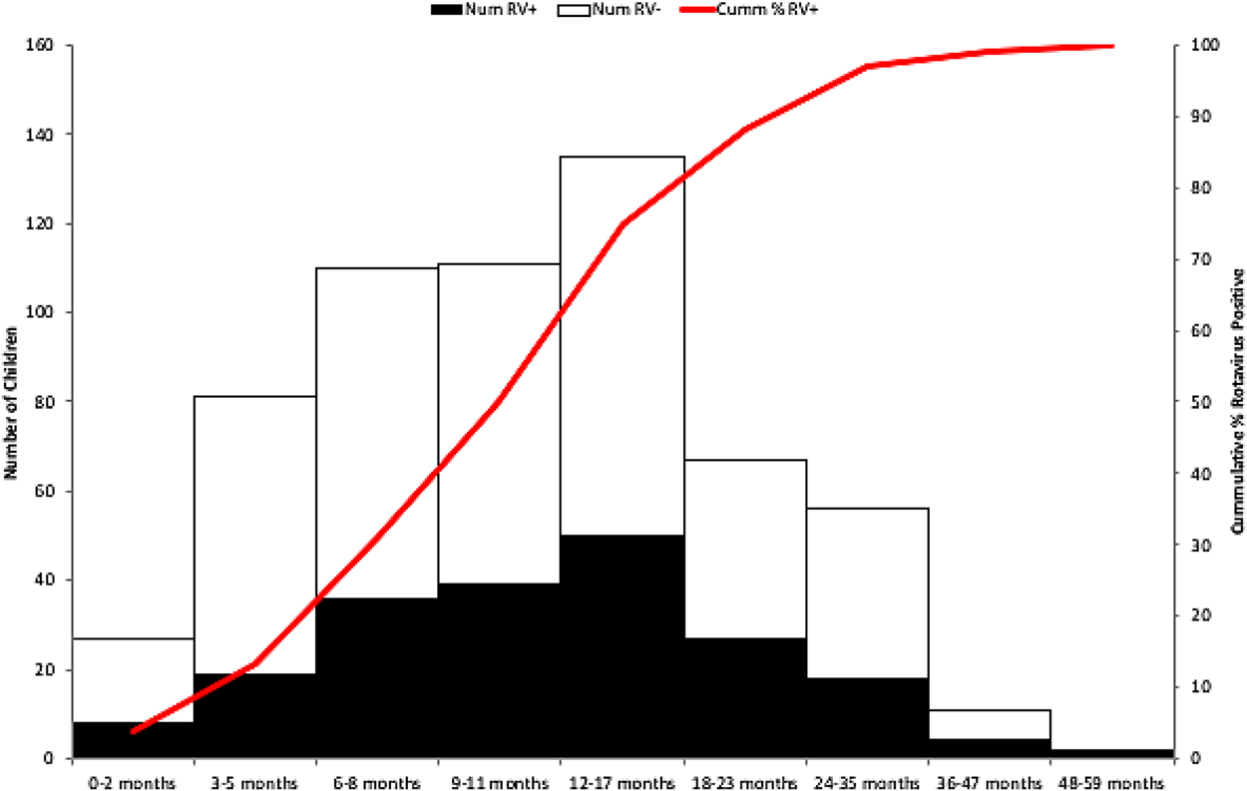
Age distribution of children enrolled in the study, by rotavirus stool positivity. Num RV+ - number of children with rotavirus positive stools; Num RV− - number of children with rotavirus negative stools; Cumm %RV+ - cumulative percentage of children with rotavirus positive stools.

Of 967 children hospitalized with diarrhea that were screened for possible inclusion in the VE evaluation, 600 were enrolled from 1 September 2014 to 31 August 2017 and included in the analysis (Fig. 1). Of the 600 enrolled children, 203 (34%) were RV positive. The median age of enrolled children was 11 months. Most (88%) of RV positive cases were seen in children <24 months old (Fig. 2). Although statistically significant differences (median height, weight, mid-arm circumference and possession of mattress) were seen among RV positive and RV negative children, these differences were not considerable (Table 1).

**Table 1:** Sociodemographic characteristics of all enrolled children by rotavirus test result, September 2014 – August 2017.

	Rotavirus Positive	Rotavirus Negative	p-value
	n = 203 (%)	n = 397 (%)	
Median age in months (range)	11 (1–51)	11 (1–47)	0.07
Median current weight in kg (range)	9.0 (0.9–18.2)	8.5 (0.6–20.0)	0.04
Median current height in cm (range)	71.2 (45.2–99.7)	68.5 (43.2–95.3)	0.01
Median mid-arm circumference in cm (range)	16 (12–25)	16 (12–24)	0.003
Male (yes)	116 (57%)	233 (59%)	0.72
Household enrolled in NHTS (yes)	33 (16%)	60 (15%)	0.71
Median number of people in household (range)	5 (3–14)	5 (3–15)	0.09
Median number of children <5 yrs in hh (range)	1 (1–4)	1 (1–5)	0.19
Mother’s current age (range)	29 (16–47)	29 (15–47)	0.33
Mother marital status			0.22
Single	5 (2%)	20 (5%)	
Married	101 (50%)	207 (52%)	
Co-habitation	97 (48%)	170 (43%)	
Mother education level			0.28
Primary school	45 (22%)	90 (23%)	
Secondary school	131 (65%)	246 (62%)	
Post-secondary	15 (7%)	21 (5%)	
University or above	12 (6%)	40 (10%)	
Father education level			0.20
Primary school	48 (24%)	111 (28%)	
Secondary school	123 (61%)	229 (58%)	
Post-secondary	21 (10%)	26 (7%)	
University or above	11 (5%)	31 (8%)	
Household has electricity (yes)	174 (86%)	341 (86%)	0.95
Source of household drinking water			0.18
Bore hole	5 (2%)	5 (1%)	
Covered well	7 (3%)	16 (4%)	
Open well	2 (1%)	8 (2%)	
Shared community tap	104 (51%)	235 (59%)	
Tap to house	85 (42%)	133 (34%)	
Household possessions (yes)			
Radio	168 (83%)	321 (81%)	0.57
Mattress	123 (61%)	183 (46%)	<0.001
Car	1 (1%)	5 (1%)	0.37
Television	31 (15%)	71 (18%)	0.42
Bicycle	0 (0%)	3 (1%)	0.21
Mobile phone	166 (82%)	340 (86%)	0.22
Refrigerator	0 (0%)	6 (2%)	0.08
Motorcycle	5 (2%)	4 (1%)	0.17
Computer	0 (0%)	1 (0.3%)	0.47

**Figure 3:**
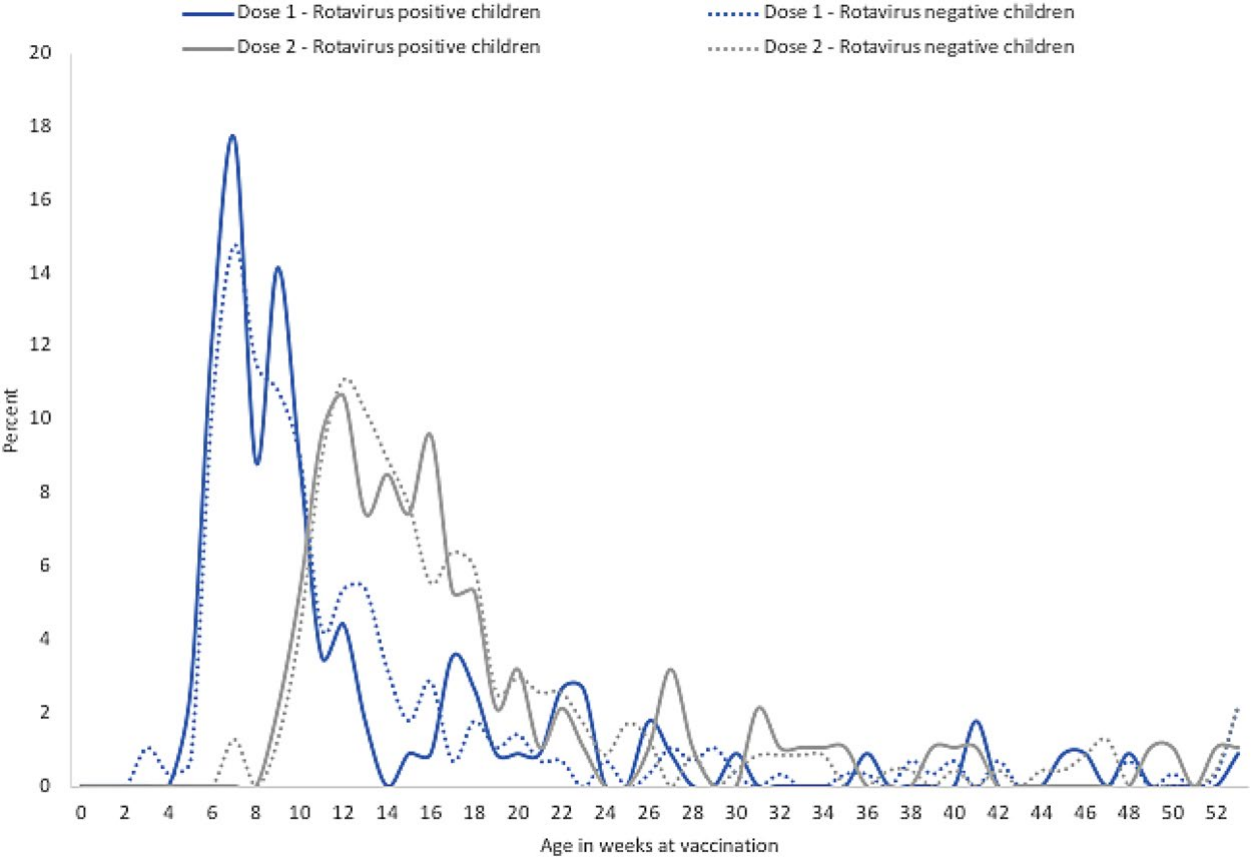
Age at receipt of rotavirus vaccine doses of children, by rotavirus stool positivity.

RV positive children were more likely to have severe disease, i.e., more diarrhea (p < 0.001), vomiting (p = 0.01) and fever (p = 0.01) than RV negative children. Likewise, the median Vesikari score was higher among RV positive (p < 0.001) but the difference was not substantial in the two groups (Table 2). 486 (81%) of the 600 enrolled children received any vaccine. The proportion of children with no RV vaccination was significantly higher among RV positive (29%) compared to RV negative (16%) children (p = 0.004). Although the EPI schedule allowed children to receive RVV up to 2 years of age, there was no substantial difference in the age by which RV positive and RV negative children received the RVV doses (Fig. 3).

**Table 2:** Clinical characteristics and vaccination of all enrolled children by rotavirus test result, September 2014 – August 2017.

	Rotavirus Positive	Rotavirus Negative	p-value
	n = 203 (%)	n = 397 (%)	
Duration of diarrhea			0.36
0 days	43 (21%)	100 (25%)	
1–4 days	151 (74%)	272 (69%)	
5 days	3 (2%)	13 (3%)	
≥6 days	6 (3%)	12 (3%)	
Max number of diarrhea episodes in 24 hours			<0.001
1–3 episodes	30 (15%)	89 (22%)	
4–5 episodes	89 (44%)	228 (57%)	
≥6 episodes	84 (41%)	80 (20%)	
Vomiting (% yes)	158 (78%)	271 (68%)	0.01
If yes, duration of vomiting			0.03
0 days	38 (24%)	58 (21%)	
1 day	85 (54%)	159 (59%)	
2 days	25 (16%)	22 (8%)	
≥3 days	10 (6%)	32 (12%)	
If yes, max number of vomiting episodes in 24 hrs			0.69
1 episode	138 (87%)	243 (90%)	
2–4 episodes	18 (11%)	24 (9%)	
≥5 episodes	2 (1%)	4 (1%)	
History of fever (% yes)	162 (80%)	277 (70%)	0.01
Temperature at presentation			0.05
≤37 °C	41 (20%)	120 (30%)	
37.1–38.5 °C	109 (54%)	195 (49%)	
38.5°-<39 °C	41 (20%)	59 (15%)	
≥39 °C	12 (6%)	23 (6%)	
Received ORS before admission (% yes)	12 (6%)	28 (7%)	0.60
Condition on arrival			0.63
Well, alert	1 (0.5%)	1 (0.3%)	
Restless, irritable	202 (99.5%)	396 (99.7%)	
Lethargic or unconscious	0 (0%)	0 (0%)	
Sunken eyes (% yes)	8 (4%)	14 (4%)	0.80
Child’s thirst status at admission			0.35
Drank normally, not thirsty	9 (4%)	25 (6%)	
Thirsty, drank eagerly	194 (96%)	372 (96%)	
Drank poorly, not able to drink	0 (0%)	0 (0%)	
Child’s skin turgor at admission			0.31
Goes back quickly (immediately)	203 (100%)	395 (99%)	
Goes back slowly (1–2 seconds)	0 (0%)	2 (1%)	
Goes back very slowly (>2 seconds)	0 (0%)	0 (0%)	
Received IV fluids during hospital stay (% yes)	202 (99%)	396 (100%)	0.16
Hospitalized	203 (100%)	397 (100%)	–
Median length of stay in days (range)	3 (1–9)	3 (1–14)	0.65
Vesikari Score			0.07
≤10 (mild)	138 (68%)	303 (76%)	
11–14 (moderate)	64 (32%)	91 (23%)	
≥15 (severe)	1 (1%)	3 (1%)	
Median Vesikari Score (range)	10 (6–15)	9 (4–17)	<0.001
Child received any vaccine			0.10
Yes	157 (77%)	329 (83%)	
No	46 (23%)	68 (17%)	
Rotavirus vaccine coverage among children who received any vaccine	N = 157	N = 329	0.004
0 Dose	45 (29%)	52 (16%)	
1 Dose	20 (13%)	44 (13%)	
2 Doses	92 (59%)	233 (71%)

Among children ≥8 months of age who were age-eligible to receive rotavirus vaccine, the adjusted vaccine effectiveness (VE) of at least one dose of RVV against rotavirus diarrhea hospitalization was 60% (95% Confidence Interval, CI: 24%, 79%) and against severe rotavirus diarrhea hospitalization, i.e. those with Vesikari score ≥11, VE was 64% (95% CI: 11%, 85%) (Table 3). The adjusted VE of at least one dose of RVV was higher among children aged 8–11 months (85%, 95% CI: 53%, 95%), compared to children aged 12–23 months (66%, 95% CI:2%, 88%).

**Table 3:** Rotavirus vaccine effectiveness among ever vaccinated children ≥8 months of age.

	Rotavirus positive	Rotavirus negative	Unadjusted VE (95% CI)	Adjusted VE* (95% CI)
	n (%)	n (%)		
Children ≥8 months	n = 118	n = 221		
0 doses	29 (25%)	25 (11%)	ref	ref
1 dose	11 (9%)	19 (9%)	50% (−25%, 80%)	40% (−66%, 78%)
2 doses	78 (66%)	177 (80%)	62% (31%, 79%)	62% (26%, 80%)
Any dose	89 (75%)	196 (89%)	61% (29%, 78%)	60% (24%, 79%)
Children 8–11 months	n = 39	n = 85		
0 doses	17 (44%)	10 (12%)	ref	ref
1 dose	3 (8%)	9 (11%)	80% (10%, 96%)	74% (−49%, 95%)
2 doses	19 (49%)	66 (78%)	83% (57%, 93%)	86% (55%, 95%)
Any dose	22 (56%)	75 (88%)	83% (57%, 93%)	85% (53%, 95%)
Children 12–23 months	n = 67	n = 102		
0 doses	11 (16%)	9 (9%)	ref	ref
1 dose	7 (10%)	9 (9%)	36% (−139%, 83%)	48% (−138%, 89%)
2 doses	49 (73%)	84 (82%)	52% (−23%, 81%)	67% (2%, 89%)
Any dose	56 (84%)	93 (91%)	51% (−26%, 81%)	66% (−2%, 88%)
Children ≥12 months	n = 79	n = 136		
0 doses	12 (15%)	15 (11%)	ref	ref
1 dose	8 (10%)	10 (7%)	0% (−232%, 70%)	−4% (−290%, 72%)
2 doses	59 (75%)	111 (82%)	34% (−51%, 71%)	39% (−52%, 75%)
Any dose	67 (85%)	121 (89%)	31% (−57%, 69%)	35% (−59%, 74%)
Children w Vesikari score ≥11	n = 39	n = 221		
0 doses	12 (31%)	25 (11%)	ref	ref
1 dose	4 (10%)	19 (9%)	56% (−58%, 88%)	15% (−267%, 80%)
2 doses	23 (59%)	177 (80%)	73% (39%, 88%)	67% (17%, 87%)
Any dose	27 (69%)	196 (89%)	71% (36%, 87%)	64% (11%, 85%)

^*^Adjusted for month/year of birth, month/year admission, and district where child lives.

## Discussion

Our findings confirm that RV is an important cause of diarrhea in the Philippines, responsible for 34% of hospitalized diarrheal cases, predominantly affecting young children. Further, we found that the monovalent RVV is effective against RV diarrhea in the Philippines, a lower middle income country in Asia. This supports our earlier findings of the substantial decline of RV diarrheal diseases in Agusan del Sur, where RVV was introduced^10^. Although lower VE was identified in older children in our study and may suggest waning effectiveness, it is also possible that unvaccinated children acquire infection earlier and are no longer susceptible to clinically significant infection because of natural acquired immunity^11^.

However, there are limitations that may have affected our results. First, RVV was not consistently available. After selective RVV introduction in September 2012 to infants from the poorest quintile, RVV was not available for nine months from October 2013 to June 2014 and then again for 11 months from June 2016 to April 2017. These prolonged periods may have precluded the identification of additional benefits such as indirect protection, which has been seen in other countries^12–14^. Furthermore, other findings such as changes in the rotavirus cyclical activity and age of RV infection were not identified. Coincidentally, during the period when vaccine stockouts in 2016 to 2017 occurred, a rise in the proportion of RV positive cases subsequently followed. This coincidental finding adds support to our results. Second, the health care system in the Philippines allows patients to access care anywhere, including the private healthcare system. Hence, it is likely that we were not able to capture all cases of diarrhea in Agusan del Sur. However, patients who go to the private health sector for management of diarrhea would also most likely obtain vaccine from the private sector. Third, unequal ascertainment of vaccination status may impact our results. Because immunization records in the Philippines are maintained in immunization registries that are kept in the public health centers, we had to visit all health centers to confirm all immunization information (obtained by review of cards or by recall). We had to exclude 20 children whose immunizations were unconfirmed to avoid misclassification. Stool test results were unknown to the study staff who collected vaccination information and it is unlikely that RV stool positivity influenced ascertainment of vaccination. Fourth, testing was not available locally so specimens had to be brought to RITM for testing. Fourteen specimens were lost in transit and could not be traced. Fifth, 298 children were unable to provide a stool specimen for testing, either because they were admitted on a weekend or they no longer had considerable stool output at the time of presentation. As these may have equally affected RV positive and RV negative children, it is unlikely that this resulted in bias. Sixth, since this is a hospital-based study, we did not detect cases and deaths due to RV that may have occurred in the community. Few deaths were identified in the study; all occurred among RV negative cases. A previous records review that we conducted was unable to quantify diarrheal deaths due to limitations in civil registration in the area where the study was conducted^10^. Lastly, observational studies are subject to limitations primarily due to the non-random allocation of vaccines resulting in possible differences in the health-care seeking behavior of the cases from the controls^15^. However, the controls used in the study have similar health-seeking behavior as the cases. The test negative design applied in our evaluation has been shown to be an efficient design useful particularly in countries with limited resources^16^ with comparable results to traditional case-control studies^16^ and has been validated against Phase 3 clinical trial results of RVV^17^.

The results of our study support the inclusion of RVV in the Philippines’ EPI. Aside from being provided in two regions in the Philippines, RVV is also available in the private sector. The EPI estimates that children who are brought to the private sector for immunization constitute less than 10% of the population. A decision on nationwide vaccine introduction should consider evidence of disease burden, cost and cost-effectiveness, and operational factors.

## Methods

### Study site

The vaccine effectiveness evaluation was conducted in D.O. Plaza Hospital (DOPH), one of the rotavirus sentinel surveillance hospitals in the Philippines. DOPH is a secondary hospital with a 100-bed capacity, located in Prosperidad, Agusan del Sur. In 2011, Agusan del Sur’s population was 808,500 with 15,946 births. The infant death rate was 8.78 infant deaths per 1000 live births and under 5 mortality rate was 1.10 per 100,000.

In the EPI schedule, children at least 6 weeks of age were eligible to receive the first dose of RVV. The second dose was given at least 4 weeks after the first RVV dose or at the same time as the Pentavalent vaccine, pneumococcal conjugate vaccine (PCV) and oral polio vaccine (OPV), as long as the child is not over 2 years old. RVV was provided to infants in the poorest quintile in Agusan del Sur starting in September 2012. In January 2013, RVV availability was expanded to all age-eligible children in two municipalities in Agusan del Sur, San Francisco and Prosperidad, regardless of socioeconomic status. In July 2014 vaccination was further expanded to all age-eligible children in the whole province. Vaccine stock-outs occurred in October 2013 to June 2014 and from June 2016 to April 2017.

### Vaccine effectiveness case-control evaluation

We conducted a test-negative case-control evaluation within the rotavirus surveillance platform of DOPH. Children aged <5 years who underwent treatment for acute diarrhea in DOPH were included in the surveillance. Acute diarrhea was defined as the passage of three or more loose or watery stools within a 24-hour period for ≤14 days. Case-patients were children who were enrolled in the active surveillance platform, tested positive for RV by ELISA, and were age-eligible to have received RVV. Controls were children who were enrolled in the active surveillance platform, tested negative for RV by ELISA, and were age-eligible to have received RVV. Stool specimens were collected and shipped frozen to the Department of Virology of the Research Institute for Tropical Medicine (RITM), where specimens were tested. Information on receipt of RVV from the immunization cards and from parents’ recall were confirmed in the public health centers’ immunization registries. If the child was not in the immunization registry and may not have received vaccine, this information was confirmed by a visit to the child’s domicile, if known, by the health worker. Children whose identity could not be confirmed by the community health workers were excluded.

### Sample size calculation

We assumed that 30% of diarrhea cases were due to RV, to detect a 60% vaccine effectiveness (VE), 90% vaccine coverage, at a 1 case to 2 controls ratio, power of 80% and 5% significance, at least 327 children were required, including 109 case-patients and 218 test-negative controls.

### Data management and statistical analysis

Data were collected from the patient’s medical charts and caregiver and provider interviews and were recorded in paper forms. Aside from surveillance information (age, RV vaccination history, address), socioeconomic status, receipt of other EPI vaccines and disease severity indicators were collected. To assess the severity of the diarrheal illness, Vesikari scoring was performed by one researcher (JET). These were then transcribed into the web-based Rotavirus Surveillance Reporting System (RvSRS ver 1.0), developed by the WHO Regional Office for the Western Pacific.

Analyses were performed using SAS version 9.4. Age, receipt of RVV doses and other vaccines as well as clinical and socio-economic characteristics were tabulated and compared between test-positive cases and test-negative controls using chi-square or Fischer’s exact test, for sparse data. Like other analyses of RV effectiveness, calculation of VE was limited to children ≥8 months of age to exclude very young children not age-eligible for the vaccine or those children whose RV vaccination may have been delayed. Unconditional logistic regression controlling for month and year of birth, month and year of admission, and district where child lived was used to calculate the odd ratio for rotavirus vaccination for rotavirus-positive cases vs. rotavirus-test negative controls. VE was calculated using the formula:

$$VE=(1-odds\,ratio)\times 100 \% $$

where the odds ratio is the adjusted odds ratio for the rotavirus immunization rate among case-patients compared with controls. All p values and 95% confidence intervals (CI) were interpreted in a two-tailed manner and statistical significance was set at p < 0.05.

### Ethics and Informed Consent

The evaluation was reviewed and approved by the University of the Philippines Manila Research Ethics Board (UPMREB 2014-167-01). Additional ethical approval was obtained from the WHO Regional Office for the Western Pacific Ethical Review Committee (2014.9.PHL.1.EPI). The study was conducted in accordance with the Philippines’ National Ethical Guidelines for Health and Health Related Research. Informed consents were obtained from all guardians of study participants.

## Data Availability

Datasets analysed in this study are available from the corresponding author on reasonable request.
